# Essays and Dissertations on Points of Medicine, Surgery, and State Medicine

**Published:** 1835-04-01

**Authors:** 


*Aufsätze und Abhandlungen aus dem Gebiete der Medicin, Chirurgie, und Staatsarzneikunde.* Von Dr. Joh. Nep. Rust. *Erster Band. Mit drei lithographirten Tafeln.*—Berlin, 1834.

*Essays and Dissertations on Points of Medicine, Surgery, and State Medicine.* By Dr. Rust. *Volume the First. With three Lithographic Plates.*—Berlin, 1834. 8vo. pp. 475.

It is not an uncommon mistake, we believe, to imagine that the medical writers of Germany are so strongly tinged with the idealism which characterizes a large portion of their general literature, that a sober Briton might almost as well read Kant or Fichte, as Hufeland or Jörg; and many persons, who possess every qualification for judging of the German doctors, save the having perused their works, give very broad hints that it is impossible to understand our Teutonic brethren, and that it would be useless, could it be done. We trust that the analyses of German books which we have given from time to time may have served to disabuse many of this unwholesome prejudice, by showing them that Beck and Kramer, and Moritz Strahl, and Hohl, are as plain and sensible as if they were doing a large practice in the cities of London and Westminster.

The work of which the first volume is before us, is another one to be added to the praiseworthy list: it is the production of one of the most distinguished practitioners in Germany, who, having now arrived at the sixtieth year of his honourable and well-spent life, thinks it better to collect his choicest essays into one harmonious whole, than to leave the task to tardy, and perhaps ill-judging executors.

The present volume contains the commencement of an account of Dr. Rust's practice, when surgeon to the Vienna Hospital, from November 1, 1810, to May 31, 1815; a period of four years and a half, during which he treated 3889 cases. At the end of the volume are three essays: 1, on magnetism, as it was carried on at Vienna; 2, on the influence of diet and regimen on the sick; 3, on clinical instruction.

It would be vain to attempt an analysis of so condensed a book as Dr. Rust's, forming in fact the first part of a system of surgery derived from actual practice, and we must therefore content ourselves with giving a few of the more interesting passages, and referring our readers to the work itself for further instruction.

The following observations on the use of plasters are remarkably judicious:

"This remedy is far too much neglected in modern surgery, which more frequently employs inunctions, which in many cases are of inferior efficacy. A plaster is a powerful remedy, and yet its healing powers are usually better known to old women and quacks than to physicians. This superiority to inunction partly depends on the plaster being a more permanent application; but there are other circumstances on the same side of the question to be taken into account, provided the plaster be properly applied. The fault, however, is often committed of judging of its powers as a remedy from the materials of which it is composed; and if these powers do not agree with the principles laid down by writers on the Materia Medica, the remedy is rejected as inefficacious, without considering that here the form in which the remedy is applied produces the effect rather than the medicinal substance, and that, through the various additions which the physician thinks himself obliged to make to the ordinary plaster mass, it ceases in fact to act as a plaster.

"The essential effect of a plaster is derived from its acting as a cover through which perspiration cannot pass; and thus it causes a continual irritation of the skin, while it protects the diseased part from all injurious external impressions. It is through these properties alone that it becomes an important remedy, and the better it is adapted to answer these views, the more efficacious it is usually found to be. If then a plaster is to act, it must be prepared of a strongly adhesive, and not easily penetrable or soluble mass; it must be spread upon thick linen, or, what is better, upon thin leather; and it must not be too small, but, on the contrary, be larger by the breadth of a finger than the diseased part which it is to protect; nor is it to be changed without necessity, but must rather remain till it comes off spontaneously. It is not the medicinal substance of which the plaster is composed, but the equable temperature in which the morbid part is kept, the animal vapour which collects under the impenetrable covering, continually secreted and again absorbed, and the permanent stimulus to the skin, exciting not merely the diseased, but also the neighbouring healthy vessels, into constant action, which produce those beneficial consequences, and particularly those resolvent and dissolvent powers whose effects we so frequently have the opportunity of observing after the application of plasters. Hence it appears why it is exactly those pharmaceutical combinations, (particularly the herb-plasters,) to which, according to the laws of the Materia Medica, the greatest discutient virtue is ascribed, that have this virtue the least, and therefore the physician who, in pursuance of his theories, mixes conium, melilotum, &c. with the ordinary plaster mass, is disappointed in his expectations; for these remedies, when externally applied, have nothing medicinal but their bad smell, and destroy the adhesiveness of the plaster, on which its other qualities depend. And hence, too, it appears why any domestic plaster, made of resin and other adhesive substances, will often remove a swelling in a short time, on which ointments, and liniments, and plasters enriched with vegetable and mineral substances, have been exhausted in vain.

"In obedience to this conviction, the fruit of experience, I have for more than twenty-five years used only the following plasters: the gummy diachylon, the common mercurial plaster, or the one compounded with camphor and opium, and the ammoniac plaster prepared with vinegar; and I can state, that, with nothing but these applied as directed above, I have resolved and healed indurations in membranous, glandular, and bony structures, which had obstinately resisted all other remedies." (P. 28-31.)

Thirty-four cases of burns occurred; twenty-eight of the patients were discharged cured; one, who had been burnt with gunpowder, became incurably blind, and was transferred to the workhouse *(Versorgungshaus)*; four died, and one remained under treatment. Dr. Rust ascribes the fatal termination in cases of burns to a loss of the function of the skin, and would add the skin-death, to the heart-death, brain-death, and lung-death mentioned by Bichat. His treatment is that of most sensible practitioners. In ordinary cases he thinks cold water the best remedy; but, if the burn be very extensive, it cannot be employed with propriety, lest it should still further depress the function of the skin. In these severer cases, it is necessary to protect the terminations of the nerves laid bare by the destruction of the epidermis; and this is best done by soothing applications, such as cream, butter, or oil. A mixture of linseed oil and lime-water is particularly good, but care must be taken that it does not spontaneously ignite. Dr. Rust has also frequently used Stahl's salve with the most satisfactory results, especially in cases where deformity was to be guarded against; it consists of equal parts of melted butter and yellow wax repeatedly rubbed down with water. Instead of this, he sometimes used an ointment composed of butter, yolk of egg, and linseed oil. When suppuration took place, saturnine remedies were avoided as much as possible, partly because they cannot be borne, and partly because they cause deformed scars. Dr. Rust never tried the use of oil of turpentine, brandy, or similar stimulants, and attributes the apparent success which has sometimes attended their use to Nature, who is able to overcome not only the disease but the remedy.

It is painful to see the frequency with which sarcasms of this kind are bandied about, even among the most calm and philosophic practitioners; and it is equally painful to reflect how many years must pass away before these epigrams can be answered by facts; before the best treatment of severe burns, for example, can be demonstrated by what M. Louis calls the numerical method, and we can know whether we ought to rely upon Kentish or upon Rust.

Later experience has taught our author the advantage to be derived in some cases from repeatedly sprinkling flour on the burnt surface, from the application of cotton, or scraped potatoes, and lastly from the use of a solution containing one or two grains of nitrate of silver to the ounce of water. The last remedy deserves our especial attention, partly on account of its desiccating quality, and partly because it forms as it were an artificial epidermis, and thus protects the skin from the action of the atmosphere.

Among the cases of burning our author includes one of poisoning by sulphuric acid. The patient's life was saved, but a difficulty of swallowing remained behind, which was more troublesome some years subsequently than immediately after her recovery, and compelled her again to seek for advice.

"Inflammation of the tongue occurred twice; once in consequence of the tongue having been violently compressed between the teeth during convulsions; and once it accompanied a severe salivation, caused by less than six grains of calomel. The tongne dry, hard, and inflamed, projected beyond the lips like an immovable mass, the saliva flowed incessantly from the mouth, and the patients were without sleep, and in a state of the most utter anxiety and disquietude.

"Large general bleedings, leeches to the chin, a blister to the back of the neck, emollient waters held in the mouth, steamings, and vegetable juices applied with a brush, (*Pinselsäfte*,) resolved the inflammation in the second case; but, in the first one, they produced a scarcely perceptible alleviation of the sense of suffocation, until, on the fourth day of the disease, three deep incisions were made into the tongue, evacuating a large quantity of blood, and causing an immediate diminution of the swelling. On the following day, the tongue, which was half its previous size, no longer protruded beyond the lips, and hardly a trace was to be seen of the incisions. Two deep incisions were now effected more backwards, towards the root of the tongue, as the bistoury could now be introduced farther into the cavity of the mouth; and thus this violent and unfrequent inflammation was so perfectly dissipated under the continued use of mucilaginous and light aromatic mouth-waters, that not a trace remained of the diseased metamorphosis of an organ which had been so seriously affected." (P. 58-59.)

Ninety-three cases of bubo occurred, chiefly syphilitic; eighty-seven patients were cured, two discharged at their own request, one was transferred to a different division of the hospital, and three remained under treatment. The bubo was usually a secondary or consecutive form of disease, more rarely primitive, and still more seldom sympathetic. Dr. Rust's practice will appear eccentric to most of our readers; for it was only when the disease appeared in the last-mentioned form, that he attempted to discuss the tumour: in the majority of cases he encourages the suppuration. He is of opinion that the principle which has been so confidently laid down, that buboes are to be discussed, has done as much mischief as the local treatment of gonorrhœa by injections; and would have done much more, were it not that the precept is very difficult to be put into practice. He asserts that these erroneous rules have been derived from the fact that a bubo, which has been opened too early, or which has been suffered to burst too late, is one of the most obstinate of diseases; but thinks that the difficulty is entirely got over if we open the bubo as soon as all induration has disappeared around the swelling, and no sooner.

Calomel and the Ung. Hydr. are the mercurial preparations which he prefers in this form of syphilis, and he declares, from repeated experience, that the corrosive sublimate is not only unsuitable, but injurious.

Dr. Rust narrates at great length a case which had nearly terminated fatally. The patient, a young man, of twenty-three, entered the hospital, June 21st, 1814, with a suppurating bubo, and all the signs of syphilitic cachexia. The bubo had been opened by caustic far too early, for the surrounding parts were as hard as a stone. He had already been ill four months, and got progressively worse for several more, under a great variety of treatment, when, a consultation being held, it was almost unanimously determined that he was suffering not from syphilis, but from mercury. Sulphur baths and other remedies were now ordered, but without advantage, and, at the end of December, the surface of the sore was ash-coloured, putrid, slimy, ichorous, and stinking.

Dr. Rust, having often observed that, when it is desirable to alter the character of a sore, the nitrate of silver, corrosive sublimate, and the red precipitate, are the most efficacious applications, began with the first two, but with little or no benefit. He then tried the red precipitate, with the most marked advantage: the sore lost its ashy hue, became perfectly clean and red, and secreted healthy pus. Still it made no progress in healing; and Dr. Rust, believing that the syphilitic virus was not yet extinguished, and having found such remarkable advantage in the external use of the red precipitate, determined to prescribe it internally. On the 13th of February, 1815, he ordered one sixth of a grain of red precipitate with five grains of sulphuret of antimony, and five of sugar, to be taken twice a day.

"If the effect of the red precipitate, externally applied, might be called wonderful, much more did its effect, when given internally, deserve this epithet; for the patient not only bore the powder without the slightest inconvenience to the digestive organs, but his whole look was altered in a short time. What calumba, cinchona, Iceland moss, opium, camphor, musk, and ether had been unable to do, was at once effected by the powder. The colliquative sweats and diarrhœa ceased; the patient's appetite returned, his strength visibly increased, the surface of the sore daily diminished, and, what had been aimed at in vain in a period of eight months, (or, to count from the beginning of the disease, in a year,) was now perfectly attained in six weeks. On the 30th of April, 1815, the patient was dismissed perfectly cured, after having taken twenty-three grains of the red precipitate.

"Since this period I have met with several cases of highly neglected, ill-treated, suppurating and gangrenous buboes; and, in every instance, the red precipitate sprinkled on the sore has shown extraordinary power in changing its character." (P. 74-75.)

Dr. Rust adds, that the general cachexia and weakness induced by syphilis cannot be cured by cinchona or acids, or by any medicine save mercury; but makes a half exception in favour of Zittmann's Decoction, which is a sort of Decoct. Sars. C.

In treating of inflammation of the prostate, our author mentions a remedy recommended by Dr. Fischer, namely, thirty or forty grains of muriate of ammonia every two hours; and says that he has himself found it very useful in diseases of the bladder and prostate.

The number of ulcers treated during these five years was considerable, namely 688, including 65 chancres; of the remainder, 562 were in the feet, 61 in other parts. The rules laid down for the treatment of different ulcers show the practised surgeon, and form a good compendium of "Helcology," as Dr. Rust calls it. They are as follows:

1. *Irritable ulcers.* If the ulcer was painful, its bottom brown, rather dry than moist, the secretion scanty, and the edges raised and painful, and even the neighbouring parts red and inflamed, the continued application of plain lukewarm water, or Goulard water and emollient bran poultices, were the most serviceable remedies. But there are ulcers of so irritable a character, that, to touch them, excites convulsions; and leeches, applied in the vicinity of the diseased part, increase instead of lessening this sensibility. The surface of the ulcer has nothing peculiarly striking about it, not even an appearance of irritation or inflammation, and is commonly far from large; it is sometimes smooth, shining, and devoid of all granulation, and at others covered with pale loose cellular substance. This rare species of ulcer has several times been observed by Dr. Rust, who affirms that there is only a single remedy of efficacy in these cases, but that is a sure one, namely, the red precipitate.

2. *Fetid ulcers.* In unclean sores, with very fetid secretion, and putrid surface, which was even in part sphacelous, the application of charcoal, of chamomile flowers, of myrrh and of camphor, (sometimes in powder, and sometimes made into an ointment with oil of turpentine,) pyroligneous acid, camphor wine, and solution of chlorine, were the most efficacious means of removing the fetor, and of chemically altering and improving the secretion; at the same time that they strengthened the relaxed fibres, and by increasing the activity of the vessels, they cleaned the sore, and threw off the slough.

3. *Luxuriant granulation.* When the surface of the ulcer was luxuriant, but not otherwise unhealthy, the best effects were produced by the continued application of cold water, and Goulard's solution, with low diet, frequent aperients, and an elevated position of the suffering part. When the granulation was luxuriant, and at the same time spongy, the application of the solution of nitrate of silver with opium, of a solution of corrosive sublimate (from two to three grains to the ounce), of the expressed juice of the small-leaved plantain of camphor wine, or sometimes merely a dry and rather tight bandage, were the most efficacious remedies.

4. In the case of *sluggish ulcers* warmth and moisture were applied in the shape of fomentations and bran poultices, and they were dressed with stimulating ointments, such as the Ung. Basilicum cum Tr. Myrrhæ, or the Ung. Hydr. rubr., and with distinguished success.

5. *Varicose ulcers.* In old ulcers of the foot, when the surrounding parts were varicose and callous, the edges indurated and prominent, and the bottom smooth and cup-shaped, the whole of the affected parts were wrapped up in strips of plaster, with evidently favourable results. A simple unirritating plaster, such as the Empl. Plumbi or Empl. Saponis, was always selected for this purpose. The pressure soon improved the condition of the parts, the relaxed surface of the sore was stimulated, and the indurated edges became soft. The remedies having produced their effect, their continuance would have been injurious, and other expedients were adopted for the cicatrization of the ulcer. A solution of nitrate of silver with Tr. Opii was generally used, or else Goulard's water, according as stimulants or sedatives seemed to be indicated.

6. *Gouty ulcers* were also treated with the greatest advantage by the application of a simple plaster, such as those just mentioned, though the form and condition of the sore did not always allow of its being put on circularly. Other remedies could rarely be borne, especially if they were applied in a moist or fluid form.

7. *Scrofulous ulcers* were injured by all emollient or relaxing remedies; but, on the other hand, the most efficacious were the sprinkling the secretory surface with red precipitate, chamomile flowers, bark, &c.; the application of the phagedenic water, the solution of nitrate of silver, the expressed juice of the small-leaved plantain, and the following dressings.

℞. Ung. saturnini ℥j.; Hydr. præcip. rubri ʒij.. M. ℞. Extr. Anthem., Conii, Calendulæ, *aa* ʒij.; Aquæ Laurocerasi ℥ij.; Tr. Opii ʒiss. M.

In cases which had been very much neglected, the entire removal of the edges of the sore by the scissors or knife, considerably hastened the cure of the sore itself.

8. *Scorbutic ulcers* were most effectually treated by dressing them with Theden's arquebusade, camphor wine, pyroligneous acid, and powdered charcoal.

9. *Impetiginous ulcers*, whether they took their rise from a psoric, herpetic, or any other eruption, got well under the use of mercury (in the shape of the red or white precipitate, or the sublimate,) acetate of lead, sulphate of zinc, and charcoal. In addition to the combination of lead ointment with the red precipitate given above, phagedenic water, and fomenting the part with infusion of chamomile combined with acetate of lead and tincture of opium, Dr. Rust says that he can recommend the following dressing:

℞. Ung. Saturnini ℥j.; Ung. Rosat. ℥ss.; Hydr. præcip. albi, Zinci oxydi, *aa* ʒij.; Pulv. carbon. lign. tiliæ ʒiij. M.

Suitable general remedies were of course administered at the same time; among the best were sulphur, sulphuret of antimony, the Æthiops antimonialis, and Zittmann's Decoction.

The local application of fresh cabbage-leaves and a poultice of raw potatoes were frequently of great efficacy in cleansing the sore, particularly in herpetic ulcers.

10. *Primary syphilitic ulcers*, or *chancres*, were treated on the principle that, in the majority of cases, a general mercurial course is not requisite for the radical cure; and that the chief thing necessary is locally to change the nature of the sore, and to decompose and destroy the virus which is present. For this purpose the vesicle which forms after infection or the small excoriated spot which may already be present, should immediately be touched with the nitrate of silver, to prevent the spreading of the sore, and the absorption of the virus. This is to be done only during the first three or four days, a period when patients seldom seek advice: it is not to be recommended afterwards, when the sore has spread, and is copiously suppurating; for then the suppression of suppuration, by the forming of a crust, might give rise to vicarious disorders, especially buboes. In such cases, the application of a solution of caustic potash (a grain to an ounce of water,) was of more benefit than other and similar remedies; this is the less surprising, as caustic potash confessedly possesses the power, in so remarkable a degree, of decomposing animal poisons, that ablution with this wash after connexion has proved the most decided preventive of syphilitic infection.

Dr. Rust also recommends the black wash, with the addition of opium, in the proportion of a scruple to an ounce. The solution of corrosive sublimate, the red precipitate ointment, &c., were less frequently employed, and were obviously inferior in efficacy.

To this it may be answered, of course, that syphilitic sores will heal easily with any or no treatment, and the important point to be settled is, what treatment of the chancre will most diminish the chance of secondary symptoms without injuring the constitution of the patient, or adding fresh malignity to the secondary symptoms, should they arise in spite of the remedies.

Our author has some interesting observations on these and other topics relating to syphilis in the volume before us, (p. 163-180.) He begins by repeating, in an abridged form, an instructive set of experiments which he formerly published in the fifth volume of his Journal. Thirty patients, suffering under syphilitic ulcers of the genitals, of apparently the same kind, were divided into three classes. Ten were subjected to a purely local treatment; ten others took, in addition, mercury internally, until the local disease had disappeared; and the other ten, (following Hufeland's precept,) continued the internal use of mercury, when the local disease was cured; and if salivation appeared, they then continued the remedy after its disappearance, in diminished doses, for the same length of time that it had required to cure the sore. Those recovered the soonest, in the majority of cases, who had used mercury internally also. Within a twelvemonth, only one of those who had been treated without mercury came under treatment again for secondary symptoms, without having again exposed himself to syphilitic infection. Under similar conditions, three of those who had taken mercury internally until the disappearance of the local disease were affected with secondary syphilis; and also one of the ten patients who had continued the use of mercury after the cure of the sore, and who, for the most part, had been salivated. Dr. Rust never perceived either in these or in many other cases which have since occurred to him, that the secondary symptoms are more obstinate or more severe in those who have used mercury, than in others; but he confesses that cases of organic destruction, especially of the bones, are much rarer now that the fashion of treating syphilis without mercury has become pretty generally diffused, than formerly, when people fancied that they could not cure any syphilitic affection whatever without mercury; which, moreover, was too often administered in the most absurd manner. It is not mercury in itself, however, which seems to have so pernicious an influence on the frame, but its irrational administration, and particularly its use when the diet and regimen are ill regulated.

Nor is Dr. Rust inclined to think less highly of the internal administration of mercury from the unfavourable results attending it in the experiments detailed above. He is convinced that they are to be attributed to a bad diagnosis of the cases, or to other accidental causes: but concedes, at the same time, not only that in many cases we can radically cure local syphilis, without mercury; but that mercury, though it may accelerate the cure, is by no means able to prevent the transition of the disease into general syphilis, even when used in the most orthodox manner.

The axiom laid down by Dr. Rust in his lectures has therefore, he says, remained undisturbed for twenty years: *mercury can cure syphilis in all its forms, but cannot prevent it.*

Our author observes that chancres sometimes occur, which without producing the phenomena of a general lues, resist all the usual methods of treatment, and attack the cellular substance so vigorously, that if the progress of destruction is not stopped by some most efficacious local remedies, fistulous openings into the urethra take place, and a great part of the glans is destroyed. These sores are not obviously different from the ordinary ones, but are generally situated on the frænum which they soon entirely destroy. The bottom of the sore is *lardy*, and remains so, and does not become clean under the use of any of the ordinary remedies. The sore deepens and spreads daily, and is not influenced by mercury. The method of cure consists in touching the chancre very freely with lunar caustic, and then bathing the penis for a quarter of an hour in cold water. In three days the sore will be found to have resumed its lardy appearance, when the same proceeding must be repeated. After this has been done three or four times it will be found that the sore no longer spreads, but on the contrary that its edges begin to skin over; yet even then the application of the caustic is to be continued until the last spot is cicatrized. Dr. Rust has never seen a sore of this kind, when treated in this manner, followed by secondary symptoms.

The caustic itself, however, failed in one instance. The patient was a gentleman, who had previously taken calomel, by the advice of another physician, and who went through a great variety of treatment under Dr. Rust's care, without benefit. The red precipitate sprinkled on the sore was of immediate advantage, although the ointment had been used in vain.

Our author then enters at some length into the general question as to the local treatment of ulcers, namely, whether medicinal substances or simple water be preferable. Zellenberg used no application but decoction of mallows; and Kern confined himself to tepid water. It is not to be denied, says Dr. Rust, that these doctrines lessened the abuse that had formerly been made of ointments and plasters, and restored Nature to her rights. But it is possible to have too much of a good thing, and this was clearly the case with Professor Kern's method. Dr. Rust, persuaded that the healing of ulcers was essentially the work of nature, believed at the same time that the process could be furthered by art, and published his *Helkologie*, in opposition to Kern: but, as in other medical controversies, so it happened in this, that the advocates of both sides brought forward their own successful cases, as a proof of the excellence of their treatment, and clearly showed that the unsuccessful cases of their adversaries were rendered so by malpractice; and, doubtlessly, both parties had ample opportunities of triumphing in their own success, and in their adversaries' failure. So difficult is it, says our author, even in a science which depends on experiments, to perform any which shall be elevated above doubt, and unassailable by objection! Dr. Rust, however, endeavoured to do this in the following manner. An ulcer was divided into two equal parts by a small strip of adhesive plaster; and one half of its surface was covered with linen dipped in lukewarm water, while the other was dressed with applications varying according to circumstances. This experiment was made with all the usual dressings, in all kinds of ulcers, and in a great number of cases. The patient sometimes took no medicine, at others was treated on general principles. The effect of each dressing was carefully compared with that produced by the tepid water, and the results put down. It appeared, beyond all contradiction, that the simple treatment with tepid water was sufficient in many cases, and in many was even preferable to unctuous dressings; in many, again, it was disadvantageous, particularly where the granulation was too luxuriant; and in very many cases it effected nothing, or so little, that while the other half of the ulcer was already changed into a simple suppurating sore, the part treated with plain water was hardly cleansed superficially, and, instead of plastic pus, continued to secrete fetid and virulent ichor.

Besides these results, the experiments led to a more accurate knowledge of the different efficacy of the ordinary dressings, and their substitutes. In order to investigate this more closely, two strips of plaster were placed in the form of a cross over the ulcer, thus dividing it into four parts. Suppose the ulcer to have been gangrenous or putrid, one part was treated with nitre, another with cinchona, a third with charcoal, and the fourth with oil of turpentine, or with some ointment, or with plain water. Or, if the object was rather to balance the respective merits of similar substances, than to contrast those of opposite ones, drugs of the same class were applied together; for example, the barks of cinchona, oak, and willow; or solutions of nitrate of silver, caustic potash, and nitrate of mercury. The most unexpected results were the consequence. Thus, in flabby, unclean, putrid, and sphacelous sores, the gastric juice of animals, so much recommended by Carminati and Sir Everard Home, was far less efficacious than nitre, in the highest degree of putridity, and in slighter degrees was inferior to charcoal, camphor, and oil of turpentine. Thus, too, powdered rhubarb, though recommended by Sir E. Home in torpid ulcers with a flabby but abundant granulation, was not superior to other aromatic and slightly astringent powders, such as powdered chamomile; nor to other dressings indicated in these cases, such as a solution of nitrate of silver, of corrosive sublimate, of camphor wine, &c. Cinchona, again, whether applied as a fomentation or a powder, could neither be replaced by oak bark, willow bark, nor calamus root, either in decoction or powder, but only by chamomile flowers, which were so good a substitute, that cinchona was never used any more externally. A solution of nitrate of silver could not be replaced by one of caustic potash, nor by a solution of corrosive sublimate, and only partially by a solution of nitrate of mercury; but quite perfectly, as far as regards its effect upon suppurating surfaces, by the expressed juice of the plantain. Dr. Rust is of opinion, that it is by no means a matter of indifference which one is selected out of a class of similar remedies: for example, whether we make use of cinchona or oak bark, caustic potash or nitrate of silver. If the remedy is ill selected, the cure is delayed, or perhaps not effected at all.

In a subsequent part of the work, (p. 382-397,) Dr. Rust has recorded his opinions on the nature and treatment of constitutional syphilis. One hundred and ninety-five cases of this disease occurred; of which, 170 were perfectly cured, six were discharged uncured, seven were transferred to a different division of the hospital, three were sent to asylums, *(Siechen-Versorgungshäuser,)* on account of important mutilations, six died, and three remained under treatment.

Our author says, that of all the remedies which have been substituted for mercury in the treatment of syphilis, no one has been so much used as muriatic acid, which was recommended by Zeller, in a treatise printed in 1797; and he tells us, that this writer supposed calomel and corrosive sublimate to owe their efficacy to the muriatic acid which enters into their composition. Dr. Rust took the trouble to go through the journals of the syphilitic wards in the Vienna hospital; and he found that many hundred patients, labouring under syphilis in all its forms, had been cured without taking a grain of mercury: in fact, for many years it played quite a subordinate part in these wards, and the quantity of mercury annually prescribed was a mere nothing, when compared with the number of patients, and the quantity of muriatic acid which was used. Dr. Rust accordingly resolved to use muriatic acid in the same manner; that is to say, giving a drachm daily in two pounds of barley-water, but did not succeed in curing a single patient. At first he was unable to explain so singular a discrepancy in the results; but he soon discovered that it depended on a difference in the diet. Von Zellenberg, who had had the syphilitic wards under his care, gave his patients as little as possible to eat, and Dr. Rust determined to copy him. He did so, and was successful. He then diminished the dose of muriatic acid, and ultimately omitted it altogether; and the results were equally favourable.

But, although our author fully satisfied himself that the disease might be extirpated either by simple abstinence, or by the use of sarsaparilla, (which he considers a debilitating medicine,) or by saline purgatives, with occasional blood-letting, still he thinks it unjust to found upon these experiments a condemnation of mercury, and to contest its anti-syphilitic powers, which have been confirmed by the experience of centuries. Putting aside the debilitating effects of the antiphlogistic method of curing syphilis, when long continued, it is far from being true that every case of the disease can be radically cured without mercury. This is shown, not only by cases of general syphilis, which, after having been fruitlessly treated for months and years with low diet, cleansing ptisans, aperients, nitric and muriatic acids, muriate of gold and soda, &c. were at last cured by mercury; but, in addition, by the relapses of cases apparently cured by the antiphlogistic method, which were never so frequent as since this way of treating method has become the order of the day in Germany also. Hence we ought to be content with having demonstrated that low diet and the antiphlogistic treatment are not only able of themselves to subdue syphilis in its milder forms, but are extremely valuable adjuncts to mercury.

Our author insists strongly on the necessity of the mercurial course being accompanied by a very low diet, and says, very justly, that, the more strictly this is observed, the more quietly the patient stops at home, or in the hospital, the less he is exposed to changes of temperature, or other occasions of taking cold, and, the more sparing his diet, the less mercury will be necessary to subdue the syphilis, the quicker and the more entire will be the cure of the patient, and the less shall we have to do with after-diseases, which are too commonly attributed to the mercury.

And, as it is by no means a matter of indifference under what circumstances, neither is it unimportant in what form, mercury is given. The following are Dr. Rust's opinions on the latter point.

1. Calomel is indispensable in all the forms of syphilis whose essential character consists in inflammation,—such as buboes, inflammation of the prepuce, of the testes, of the conjunctiva of the eye, &c., as well as all the forms which make their appearance in vigorous individuals, showing increased power of production by new formations. In the latter cases it should be given in large doses, the best method being Weinhold's, which is as follows: The patient is to have eight ten-grain doses of calomel, and four powders, each containing fifteen or twenty grains of jalap, and the same quantity of tartrate of potash. He is to take one dose of calomel on an empty stomach, about an hour before bedtime, and then drink two cups of broth. After the lapse of half an hour, another dose is to be taken, and then two cups more of broth. Twelve hours afterwards, the patient takes a few small cups of coffee without milk; and this is generally followed by three or four fluid evacuations. If this is not the case, one of the aperient powders is taken. The same cycle of remedies is to be repeated on the fourth, seventh, and tenth day, when the doses of calomel will have been consumed; and, if necessary, the course may then be repeated.

2. Syphilitic eruptions, chancrous sores of the neck, the nose, or the frontal sinuses; syphilitic iritis; and all the secondary forms of the diseases which quickly spread, seem to put on a carcinomatous look, and threaten the loss of any organ, demand the active use of corrosive sublimate; and, if the malady is particularly obstinate, the internal use of the red precipitate, which is best given in Berg's method, as follows:

℞. Antim. Sulph. nigri, ℈viij.; Hydr. Præcip. rubri, gr. ij.; Sacchari albi, ℈ij. M. et div. in pulv. xvj. Sumat j. omni manè et nocte.

As soon as these powders have been consumed, the prescription is repeated, but with two additional grains of the red precipitate. When these powders have been used, the quantity of the red precipitate is again increased by two grains; and so on, until the quantity of the mercurial salt in the prescription amount to ten grains. As soon as this point has been reached, the quantity is diminished by two grains in each prescription, until it sinks down to two grains again. The whole course therefore lasts nine weeks; but the desired object is often obtained by half the course, that is, by the use of the remedy in an ascending series only. Should diarrhœa occur, opium is added to the powders, and cinnamon, in case of nausea or vomiting.

3. In syphilitic affections of the bones, the ligaments, or bursæ mucosæ; in lymphatic exudations in the joints, the eye, &c.; in ulcerous metamorphoses of the surface of the skin; in cases where organic destruction is already going on, and whenever the state of the intestinal canal renders it intolerant of mercury administered internally, the ointment is to be rubbed in.

4. No remedy is superior to corrosive sublimate in rapidly improving the appearance of any syphilitic malady, or in arresting the progress of organic destruction. By itself, however, it is the least capable of effecting a radical cure, or of securing the patient against relapses.

5. Among all the methods hitherto known of curing an inveterate lues by mercury, none is less adapted to its object than Hahnemann's mercurial course; and, on the other hand, none is more adapted to do wonders in desperate cases than a methodical course of inunction and abstinence. This combination was employed, with the most distinguished success, in the Vienna hospital; and one cannot but smile, says our author, to hear objections brought forward against it by uncalled clamourers *(unberufene Schreier)*, who, without any experience of their own, endeavour from behind their desks to write down the merits of this system.

Dr. Rust is no pessimist, and acknowledges that the treat ment of syphilis has much improved of late years.

"Such cabinet pieces of syphilitic metamorphoses, and ill-treated patients, as I used to meet with continually, only eighteen years ago, here in Berlin, are never seen now; and a multitude of secondary symptoms, which formerly yielded only to abstinence and rubbing-in, are now frequently cured solely by the use of Zittmann's decoction, provided that a proper diet is observed at the same time, and that the decoction is used in the manner prescribed; so that, in fact, the cases in which the severer system is necessary occur but seldom. In such cases, however, it must be employed in the only effective manner, and no modifications must be permitted, which abolish the very essence and peculiarity of the course. It is not any and every application of mercurial ointment which may pass current for the course; and he who imagines that, by merely lowering and altering the patient's diet, rubbing in every other day, ordering a bath on the intervening day, and an aperient occasionally, that he has employed the course just mentioned, and at the same time has taught something new, merely proves that he has not comprehended in what the essence of the methodical course of inunction consists, in what is its salutary principle, and in what it differs from the irregular courses of rubbing in which have been known and put in practice for centuries." (P. 396.)

The observations on the influence of diet in the treatment of disease (p. 439-452,) are remarkably sensible; but, though much neglected in practice, there is little in them which we could present to our readers, as new in theory. Dr. Rust asks, what are the remedies by which a synocha is usually and successfully treated? and answers, that the fortunate result may be attributed, with more justice, to low diet, repose, and a proper temperature around the patient, than to the Decoctum Althææ, the Inf. Verbasci or Sambuci, a drachm of nitre, a few drachms of sulphate of soda, an ounce of plum or elder jam, the favourite Spir. Mindereri (Liquor. Ammon. Acet.), or the saline draught.

And, a couple of pages farther on, he says,

"This is the best explanation of the fact that, though diseases have been treated in the most different manner in different countries, the proportion of cures to deaths has always remained pretty nearly the same; because the dietetic regimen in different times and places has been far more uniform. In my opinion, giving the Aura Camphorata or the Inf. Valerianæ, with a few drops of Sp. Æth. Sulph. c., instead of Decoct. Althææ and nitre, is of less importance than the quality of the atmosphere surrounding the patient; or his taking, at an improper time, a bottle of wine, eating heavy and indigestible ammunition bread, a piece of roast beef, or a rich goose-liver pasty." (P. 444.)

Dr. Rust mentions, with great pleasure, the case of a lady, whom he recommended (in order, he says, to advise something that had not been advised before,) to eat nothing but spinage; and, solely by using this diet for ten weeks, she was cured of a St. Vitus' dance, which had resisted every remedy up to her twenty-second year. Hufeland mentions a case of obstinate impetiginous disease, which was cured by eating apples; whence he concludes that malic acid is a very efficacious remedy in this affection: but our author supposes, and we think with greater probability, that the merit is rather to be ascribed to the apples, as a whole, being used for food, than to this acid in particular. Dr. Rust observes, too, that the guzzling townsman would lose his gout, and the countryman his chronic diseases, by exchanging diet for a time; the former giving up his meat, the latter his vegetables.

The last essay, on Clinical Instruction, (p. 455-475,) is extremely well written; and Dr. Rust discusses, not as an advocate, but as a judge, the advantages to be respectively derived from attending hospital practice, visiting the poor at their own houses, or attending the instructions of a teacher who superintends a *Klinik*, i. e. a ward containing fifteen or twenty picked cases. To see the effects of treatment *en masse*,—to observe the powers of medicine, when it has to contend against penury as well as disease,—and to learn the practice of physic and surgery, as it were, chapter by chapter, are among the more obvious privileges of the three methods of learning.

Thrice happy is the student who combines them all! He will learn at one time what can be done with a few cheap and simple drugs, and at another he will find new tools in the most exquisite refinements of modern pharmacy; sometimes relying on his own diagnosis, he will at others have his errors (those parents of truth,) corrected by the most eminent practitioners. Such a man, neither despising the lessons to be derived from books, nor neglecting those to be conned at the bedside of the patient, will not split upon the rock of empiricism, nor be swallowed up in the vortex of book-learning. It is by the collected wisdom of the past, elaborated and improved in a congenial mind, that the great practitioner is formed; for we may almost apply to the physician what the most elegant of critics has said of the poet:

——ego nec studium sine divite venâ
Nec rude quid prosit video ingenium, alterius sic
Altera poscit opem res, et conjurat amicè.

